# A dyadic study of psychological well-being of individuals with Parkinson’s disease and their caregivers

**DOI:** 10.1038/s41598-020-79609-z

**Published:** 2021-01-13

**Authors:** Yu Lee, Yu-Jie Chiou, Chi-Fa Hung, Yung-Yee Chang, Ying-Fa Chen, Tsu-Kung Lin, Liang-Jen Wang

**Affiliations:** 1 Department of Psychiatry, Kaohsiung Chang Gung Memorial Hospital, Chang Gung University College of Medicine Kaohsiung, Kaohsiung City, Taiwan; 2 Department of Neurology, Kaohsiung Chang Gung Memorial Hospital, Chang Gung University College of Medicine Kaohsiung, Kaohsiung City, Taiwan; 3 Department of Child and Adolescent Psychiatry, Kaohsiung Chang Gung Memorial Hospital, Chang Gung University College of Medicine, No.123, Ta-Pei Road, Kaohsiung City, Taiwan

**Keywords:** Diseases, Health care

## Abstract

Parkinson's disease (PD) is an incapacitating neurodegenerative disease. Patients with PD and their caregivers may have interactive effects on each other’s psychological well-being. This study aimed to assess the dyadic dynamics of resilience, fatigue, and suicidal ideation on the depression severity of PD patients and their caregivers. In total, 175 PD patients and 175 caregivers were recruited at a medical center from August 2018 to May 2020. Structural equation modeling (SEM) was used to examine the actor/partner effects on the psychological well-being of both the PD patients and their caregivers. The most common psychiatric diagnoses of both the PD patients (28.6%) and their caregivers (11.4%) were depressive disorders. The PD patients’ and their caregivers’ fatigue, suicidal ideation, and lack of resilience were significantly associated with the severity of their depression, respectively. Interactive effects existed between psychological well-being of individuals with PD and their caregivers. Clinicians must be aware of, and manage, these contributing factors between PD patients and their caregivers in order to prevent them from worsening each other’s depression.

## Introduction

Parkinson’s disease (PD) is primarily a prevalent disease among elderly individuals^1^. PD is characterized by tremors, bradykinesia, rigidity, and postural instability; and affects approximately 1 million individuals in the US^2^. Besides motor disability, PD patients also exhibit non-motor symptoms such as anxiety, apathy, cognitive dysfunction, and depression^3^. The impacts of untreated depression extend far beyond mood symptoms; this includes greater functional disability, faster physical and cognitive deterioration, poorer quality of life, and increased mortality rates^4,5^. The estimated prevalence of depression among patients with PD varied widely among different studies (from 2.7 to 90%). Around 35% of these patients displayed clinically significant symptoms of depression. It is, however, difficult to point out a representative figure across the different study cohorts^6,7^. Studies using the DSM-IV’s criteria and a structured clinical interview, reported a prevalence of major depression ranging from 20 to 25% in all PD patients^8^.

With the disease’s progression, PD patients require increasingly more assistance in their everyday life, often delivered by caregivers^9^. These caregivers subsequently experience distress in the physical, mental, and social aspects of their lives^10,11^. This fact is reiterated in the findings of prior studies, which indicate that caring for a person with PD is associated with an increased risk of psychological distress, anxiety and depression^12^. Thus, clinicians should concern about the caregivers of PD patients, especially regarding their mood condition. Previous studies revealed that the rate of depression of caregivers of PD patients ranged from 14 to 35%^13,14^. Compared to studies used self-rated questionnaires to detect depression, the study used a structured clinical interview to diagnose the caregivers of PD patients showed a lower rate of depression (11.1%) among caregivers^15^.

Previous studies have investigated fatigue^16,17^, resilience^18,19^, and suicidal ideation^20,21^ in PD patients. Fatigue is characterized as a sensation ranging from tiredness to exhaustion, and is a frequent non-motor complaint of patients with PD (37–56%)^16,17^. Resilience is the ability to quickly overcome adversity and stress, as well as regain a normal psycho-emotional state^18^. Resilience correlates with lesser disability, a better quality of life in PD patients, and plays a critical role in adjusting to the disease^18,19^. The combining effect of motor disabilities, depression (and other psychiatric disorders), along with the neuropathology of PD may put these patients at risk of suicide^22,23^. The prevalence of suicidal ideation in PD patients ranged from 10.2% ~ 14.4%^20,21^. Furthermore, few studies that have detected fatigue^15,24^, resilience^25,26^, and suicidal ideation^27^ among caregivers of PD patients. One study demonstrated that caregivers of PD patients experienced daily physical health problems including: muscle strain, headaches, and fatigue (17%)^24^. One study found that resilience modulated the inverse relation between perceived stress and QOL^25^. Furthermore, resilience partially influenced the effects of social support on the mitigation of mental health symptoms^26^. In a large register-based cohort study, PD patient is associated with a higher risk of death by external causes, including an almost two-fold higher risk of suicide^27^. Furthermore, fatigue, degrees of resilience, and suicidal ideation were all associated with depression among PD patients and their caregivers in prior studies^15,22,28,29^.

The actor/partner interdependence model (APIM) is a model that simultaneously estimates the effect of a person’s own variable (actor effect) and the corresponding variable from the partner (partner effect) on an outcome variable^30^. The APIM has been widely applied for analyses of dyadic data in the social sciences^31,32^. Previous studies have examined the dyadic effects on QOL, the caregivers’ burdens, and sleep disturbances in PD patients and their caregivers^33,34^. Only one paper detected the impact of dispositional mindfulness in a stress-health model among dyads consisting of PD patients and their caregivers^35^. To our knowledge, there are no studies on the detection of the interactive effects that resilience, fatigue, and suicidal ideation for depression between PD patients and their caregivers using an APIM.

In sum, the hypothesis of this study was that interactive effects existed between psychological well-being of individuals with PD and their caregivers. The first aim of this study was to use an APIM to investigate the effects of fatigue, resilience, and suicidal ideation for depression in PD patients and their caregivers. The second aim of this study was to compare the prevalence of depression between PD patients and their caregivers using a standardized structured interview.

## Results

Of the 175 PD patients who successfully completed the study, 64% (n = 112) were males. The average age of these PD patients was 65.3 ± 9.3 years. Their mean education level was 10.5 ± 4.8 years, 90.3% were married, and 17.7% were currently employed. The average duration of disease was 8.8 ± 6.4 years (Table 1). Of the 175 caregivers that successfully completed the study, 68.6% (n = 120) were females. The average age of caregiver was 59.5 ± 12.3 years. Their mean education level was 11.4 ± 4.3 years, 86.3% were married, and 33.7% were currently employed. The average duration of caring was 7.8 ± 5.3 years (Table 2).

**Table 1:** Demographic and clinical characteristics of the patients with Parkinson’s disease (N = 175).

Characteristics	Depression N = 50	Non-depression N = 125	Total N = 175	z/x^2^	*p*
**Gender**				1.09	0.30
Male	29 (58.0)	83 (66.4)	112 (64.0)		
Female	21 (42.0)	42 (33.6)	63 (36.0)		
Age	66.0 ± 9.0	65.0 ± 9.4	65.3 ± 9.3	− 0.38	0.70
Age of onset	57.6 ± 11.1	56.3 ± 11.6	56.7 ± 11.5	− 0.61	0.54
Duration of PD	8.6 ± 5.5	8.8 ± 6.8	8.8 ± 6.4	− 0.09	0.93
**Education**				0.02	0.89
Less than high school (< 12)	21 (42.0)	54 (43.2)	75 (42.9)		
More than college (≧12)	29 (58.0)	71 (56.8)	100 (57.1)		
Years of education	10.5 ± 4.9	10.4 ± 4.8	10.5 ± 4.8	− 0.22	0.83
**Marital Status**				0.42	0.52
Unmarried	6 (12.0)	11 (8.8)	17 (9.7)		
Married	44 (88.0)	114 (91.2)	158 (90.3)		
Unemployment	46 (92.0)	98 (78.4)	144 (82.3)	4.53	0.03*
Comorbid with other diseases	33 (66.0)	84 (67.2)	117 (66.9)	0.02	0.88
**Past psychiatric history**				3.87	0.05*
No psychiatric history	37 (74.0)	108 (86.4)	145 (82.9)		
Depressive disorder	11 (22.0)	8 (6.4)	19 (10.9)		
Anxiety disorder	1 (2.0)	4 (3.2)	5 (2.9)		
Insomnia	1 (2.0)	7 (5.6)	8 (4.6)		
Suicide history	1 (2.0)	1 (0.8)	2 (1.1)	2.91	0.23
**Family psychiatric history**				1.03	0.79
No psychiatric history	48 (96.0)	119 (95.2)	167 (95.4)		
Depressive disorder	1 (2.0)	4 (3.2)	5 (2.9)		
Anxiety disorder	1 (2.0)	1 (0.8)	2 (1.1)		
Family suicide history	1 (2.0)	4 (3.2)	5 (2.9)	0.19	0.67
Anxiolytics/Hypnotics use	24 (48.0)	27 (21.6)	51 (29.1)	12.05	0.001*
UPDRS scores	43.6 ± 17.3	35.8 ± 13.6	37.8 ± 15.0	− 2.55	0.01*
H&Y staging	2.4 ± 0.7	2.1 ± 0.5	2.2 ± 0.5	− 1.96	0.05*
BHS	8.7 ± 5.2	5.0 ± 3.8	6.1 ± 4.6	− 4.33	< 0.001*
FSS	37.2 ± 17.3	23.6 ± 15.2	27.5 ± 16.9	− 4.80	< 0.001*
CDRISC	24.7 ± 9.7	32.5 ± 8.8	30.3 ± 9.7	− 4.45	< 0.001*
HAMD	15.2 ± 5.5	4.0 ± 2.6	7.2 ± 6.3	− 10.04	< 0.001*

*PD* Parkinson’s disease, *UPDRS* Unified Parkinson's Disease Rating Scale, *BHS* Beck Hopelessness Scale, *FSS* Fatigue Severity Scale, *CORISC* Connor-Davidson Resilience Scale, *HAMD* Hamilton Depression Rating Scale, *H&Y staging* Hoehn and Yahr staging, *p < 0.05.

**Table 2:** Demographic and clinical characteristics of the caregivers (N = 175).

Characteristics	Depression N = 20	Non-depression N = 155	Total N = 175	z/x^2^	*p*
**Gender**				0.43	0.51
Male	5 (25.0)	50 (32.3)	55 (31.4)		
Female	15 (75.0)	105 (67.7)	120 (68.6)		
**Age**	57.5 ± 9.3	59.7 ± 12.6	59.5 ± 12.3	− 1.57	0.12
Duration of caring	10.7 ± 6.5	7.4 ± 5.1	7.8 ± 5.3	− 2.40	0.02*
**Education**				0.06	0.81
Less than high school (< 12)	7 (35.0)	50 (32.3)	57 (32.6)		
More than college (≧12)	13 (65.0)	105 (67.7)	118 (67.4)		
Years of education	11.2 ± 3.8	11.4 ± 4.4	11.4 ± 4.3	− 0.44	0.66
**Marital status**				0.26	0.61
Unmarried	2 (10.0)	22 (14.2)	24 (13.7)		
Married	18 (90.0)	133 (85.8)	151 (86.3)		
Unemployment	12 (60.0)	104 (67.1)	116 (66.3)	0.40	0.53
Comorbid with other diseases	11 (55.0)	76 (49.0)	87 (49.7)	0.25	0.62
**Past psychiatric history**				13.23	< 0.001*
No psychiatric history	14 (70.0)	146 (94.2)	160 (91.4)		
Depressive disorder	3 (15.0)	3 (1.9)	6 (3.4)		
Anxiety disorder	2 (10.0)	1 (0.6)	3 (1.7)		
Insomnia	3 (15.0)	6 (3.9)	9 (5.1)		
Suicide history	0	1 (0.6)	1 (0.6)	0.13	0.72
**Family psychiatric history**				0.76	0.68
No psychiatric history	18 (90.0)	147 (94.8)	165 (94.3)		
Depressive disorder	1 (5.0)	6 (3.9)	7 (4.0)		
Anxiety disorder	1 (5.0)	2 (1.3)	3 (1.7)		
Family suicide history	1 (5.0)	5 (3.2)	6 (3.4)	0.17	0.68
Anxiolytics/Hypnotics use	6 (30.0)	11 (7.1)	17 (9.7)	10.60	0.001*
UPDRS of caring patients	41.8 ± 22.2	37.7 ± 12.2	37.9 ± 13.1	− 0.38	0.71
H&Y staging	2.6 ± 0.9	2.1 ± 0.4	2.2 ± 0.4	− 0.61	0.54
BHS	5.1 (0–14)	3.1 (0–15)	3.3 (0–15)	− 2.08	0.04*
FSS	33.4 ± 13.4	23.0 ± 13.4	24.1 ± 13.8	− 3.24	0.001*
CDRISC	23.4 ± 7.2	31.8 ± 7.6	30.8 ± 8.0	− 4.33	< 0.001*
HAMD	13.0 (3–26)	2.5 (0–13)	3.7 (0–26)	− 7.03	< 0.001*

*UPDRS* Unified Parkinson's Disease Rating Scale, *BHS* Beck Hopelessness Scale, *FSS* Fatigue Severity Scale, *CORISC* Connor–Davidson Resilience Scale, *HAMD* Hamilton depression rating scale, *H&Y staging* Hoehn and Yahr staging, *p < 0.05.

The results showed that 67% of PD patients and 50% of the caregivers had one or more physical illnesses. Seventeen percent of patients and 9% of the caregivers had a past psychiatric history; and 29% of patients and 9.7% of caregivers had used hypnotics in the past (Tables 1 and 2). The average UPDRS of the people with PD was 37.8 ± 15.0, and their average H&Y staging was 2.2 (± 0.5) (Table 1).

The most common psychiatric diagnoses of the PD patients were depressive disorder (28.6%), followed by rapid eye movement (REM) sleep behavior disorder (9.7%), insomnia disorder (8.0%), anxiety disorder not otherwise specified (NOS) (2.9%), and adjustment disorder (2.9%). Among the depressive disorders, the most prevalent was depressive disorder NOS (14.3%), followed by major depressive disorder (MDD) (12.0%), and dysthymia (2.3%). Of the PD patients, 55% had a psychiatric diagnosis (Table 3).

**Table 3:** Psychiatric diagnoses of patients (N = 175) and caregivers (N = 175).

MINI diagnoses	Patients N = 175	Caregivers N = 175
**Depressive disorders**	50 (28.6)	20 (11.4)
Major depressive disorder	21 (12.0)	6 (3.4)
Depressive disorder NOS	25 (14.3)	12 (6.9)
Dysthymia	4 (2.3)	2 (1.1)
Adjustment disorder	5 (2.9)	6 (3.4)
Anxiety disorder NOS	5 (2.9)	7 (4.0)
Panic disorder	2 (1.1)	1 (0.6)
Insomnia disorder	14 (8.0)	13 (7.4)
REM sleep behavior disorder	17 (9.7)	0
Others	13 (7.4)	4 (2.3)
No diagnosis	79 (45.1)	127 (72.6)

*MINI* Mini International Neuropsychiatric Interview, *Anxiety disorder NOS* Anxiety disorder not otherwise specified, *Depressive disorder NOS* Depressive disorder not otherwise specified, *REM sleep behavior disorder* Rapid eye movement sleep behavior disorder, *Others* Depressive disorder not otherwise specified history, Major depressive disorder history.

The most common psychiatric diagnoses of the caregivers were depressive disorder (11.4%), followed by insomnia disorder (7.4%), anxiety disorder NOS (4.0%), and adjustment disorder (3.4%). Among the depressive disorders, the most prevalent was depressive disorder NOS (6.9%), followed by MDD (3.4%), and dysthymia (1.1%). Of the caregivers, 27% had a psychiatric diagnosis (Table 3).

In the univariate analyses of the 175 PD patients, factors significantly associated with depressive disorders included more unemployment (92.0% vs 78.4%, *p* < *0.05*), higher UPDRS scores (43.6 ± 17.3 vs 35.8 ± 13.6, *p* < *0.05*), more habitual hypnotics use (48.0% vs 21.2%, *p* < *0.05*), greater suicidal risk (8.7 ± 5.2 vs 5.0 ± 3.8, *p* < *0.01*), higher fatigue scores (37.2 ± 17.3 vs 23.6 ± 15.2, *p* < *0.05*), and lower CDRISC scores (24.7 ± 9.7 vs 32.5 ± 8.8, *p* < *0.001*) (Table 1).

In the univariate analyses of the 175 caregivers, factors significantly associated with depressive disorders included the duration of caring (10.7 ± 6.5 vs 7.4 ± 5.1, *p* < *0.05*), psychiatric past history (× 2 = 13.23, *p* < *0.001)*, more habitual hypnotics use (30.0% vs 7.1%, *p* < *0.05*), greater suicidal risk [5.1(0–14) vs 3.1(0–15), *p* < *0.05*], higher fatigue scores (33.4 ± 13.4 vs 23.0 ± 13.4, *p* < *0.05*), and lower CDRISC scores (23.4 ± 7.2 vs 31.8 ± 7.6, *p* < *0.001*) (Table 2).

**Figure 1:**
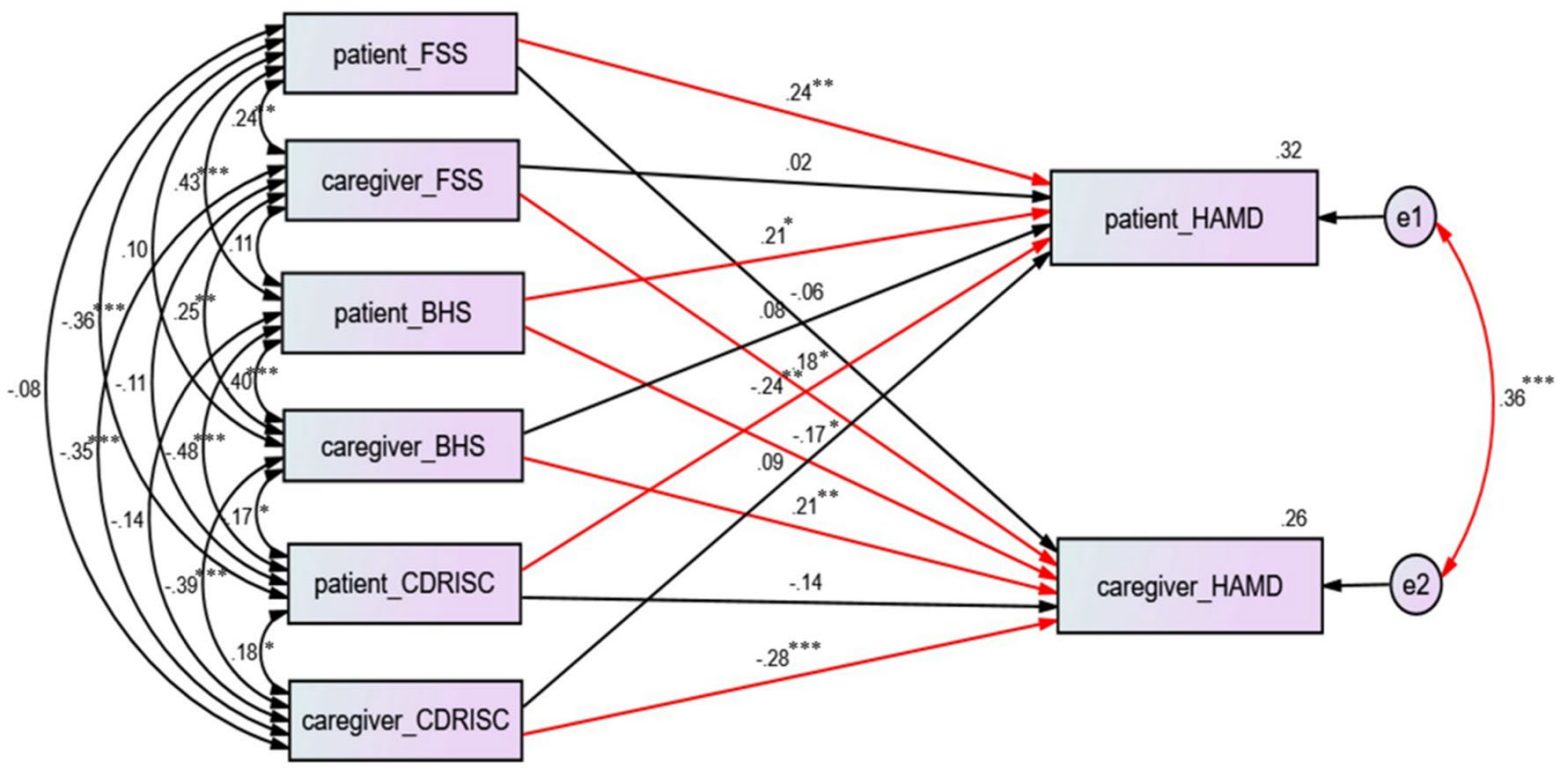
Structural equation modeling (SEM) of factors linked to depression in patients PD and their caregivers. Model summary: chi-square = 0; df = 0; p = \p. The model fit: AGFI = \AGFI; RMSEA = \ RMSEA; AIC = 72.00. *p < 0.05; **p < 0.01;***p < 0.001.

Using SEM, we found that patients’ fatigue severity (β = 0.24, p < 0.01), patients’ suicidal ideation severity (β = 0.21, p < 0.05), and patients’ resilience severity (β = − 0.24, p < 0.01) were significantly linked with depression severity in patients with PD (Fig. 1). Furthermore, we found that caregivers’ fatigue severity (β = 0.18, p < 0.05), caregivers’ suicidal ideation severity (β = 0.21, p < 0.05), caregivers’ resilience severity (β = − 0.28, p < 0.001), and patients’ suicidal ideation severity (β = − 0.17, p < 0.05) were significantly linked with depression severity in the caregivers (Fig. 1). Patients’ depression severity and caregivers’ depression severity had significant interactive effects (β = 0.36, p < 0.001).

Moreover, we conducted qualitative interview to elucidate the reasons underlying the association of suicide idea and depression among PD patients and their caregivers. Two narratives of PD patients and their caregivers recorded as follows: Patient A: “I hope that I can die soon because my health is getting worse and worse.” Caregiver A: “Once he dies, I can do anything I want.” Patient B: “I have no future. My wife cannot understand my discomfort. She doesn’t come to me at once when I feel distressed.” Caregiver B: “I think he is a whiner. His illness is not that serious. How can I take care of him if I feel downcast like him? There is a long way to go.”

## Discussion

We found depressive disorder (28.6%) is the most common psychiatric comorbidity in PD patients. However, the comorbidity rate herein is lower than which reported in previous studies on depression morbidity of PD patients (35% ~ 90%)^10,11^. Moreover, we found that depressive disorder is the most frequent psychiatric diagnosis in caregivers of PD patients(11.4%), which is lower than prior studies on depression prevalence in caregivers of PD patients (14% ~ 35%)^13,14^. The possible explanation for depression morbidity being lower, both in PD patients and their caregivers, in our results than in previous studies is that our study used structured diagnostic interviews, rather than self- rated depression questionnaires which renders lower false positive cases of depression. The advantage of using a standardized clinical interview is that it provides a clinical indicator for whether the individuals should receive proper management.

Our study found that the morbidity of depression in PD patients was twice as prevalent as in their caregivers. Patients with PD experienced motor disability, drug side effects, and cognitive impairment which might aggravate their mood condition^12^. Nevertheless, caregivers still attend PD patients through the disease process, despite having a high depression morbidity that might interfere with their care ability and quality of life^15^. Pharmacotherapy and cognitive-behavioral therapy are currently first-line treatments for depression in patients with PD^36^. Future studies are warranted to elucidate whether treatments for depression in both PD patients and their caregivers are beneficial for them each other.

We found that both PD patients’ and their caregivers’ fatigue, resilience, and suicidal ideation were associated with depression. The aforementioned clinical characteristics, which correlated with depression, were elucidated in previous studies and described in the introduction section^16–21,24–27^. In order to establish models of potential mechanisms underlie the linkage of associated factors and depression in patients with PD and their caregivers, SEM was used to examine APIM. The actor effects were as follows: patients’ fatigue, patients’ death ideation, and patients’ resilience were significantly linked with the depression severity of PD patients; caregivers’ fatigue, caregivers’ suicidal ideation, and caregivers’ resilience were significantly linked with the depression severity of the caregivers. Two partner effects were found, the first was that the patients’ suicidal ideation (β = − 0.17, p < 0.05) was significantly linked with the depression severity of their caregivers; the second was that patients’ depression severity and their caregivers’ depression severity had a significant interactive effect. Of note, we found that patients’ suicidal ideation negatively influenced their caregivers’ depression. Based on the above mentioned narratives, we can speculate that many caregivers cope with PD persons’ negative thinking and even hopelessness well. This result can partially explain why caregivers’ depression morbidity is lower than PD patients’ depression morbidity.

Based on the results of this study, we assume that caregivers of PD patients have established a high benefit finding (BF), positive life changes resulting from the struggle to cope with a stressful life event such as illness. They can overcome negative thinking from taking care of their patients^37^. Navarta-Sánchez et al.^38^ investigated factors influencing psychosocial adjustment and the QOL in PD patients and informal caregivers. They found that coping was a significant predictor of psychosocial adjustment in patients and caregivers. Macchi et al.^39^ reported that patients’ depression and quality of life contribute to caregiver burden in persons living with PD. Lindsay Penny Prizer et al.^40^ suggest that presence of a caregiver may be an important modifying variable on patient outcomes. This finding might partially support our aforementioned hypothesis.

In addition to the study of Navarta-Sánchez, a study by Karlstedt et al. (2017) tests the determinants of a dyadic relationship and its psychosocial impact among PD patients and their spouses. They found that high levels of mutuality experienced by the PD patient was associated with their QOL; and that non-motor symptoms contributed to a larger extent to the mutual relationship of PD affected dyads than motor disabilities did. However, the limited case number is the main disadvantage of this research^33^. There are few dyadic studies that use APIM analysis in different medical conditions^31,32^. This is probably the first study to examine fatigue, resilience and suicidal ideation on PD patients and their caregivers using an APIM.

The UPDRS has been the most commonly used scale to assess impairment longitudinally and disability of PD patients. The UPDRS made up of five parts with non-motor symptoms, ADL, motor symptoms, complications of therapy, and Hoehn & Yahr staging. Our results showed that UPDRS’ total score was associated with depression. We further analyzed the five parts of UPDRS and found that the score of subscales correlated to depression except on the motor symptoms subscale. Our results suggested that the motor symptoms of PD patients might be less influential on the severity of depression.

The strengths of this study are: (1) the high response rate (91.1%), (2) the use of a structured clinical interview by psychiatrists, and (3) APIM was used to clarify the interaction of PD patients and their caregivers. However, there are several limitations to this study that should be mentioned: (1) our study design involved consecutive sampling, which may have led to a sampling bias. However, a response rate of over 90% of the caregivers compromised the effect of this limitation. (2) Our samples were from a general hospital, which may not be representative of the general population. (3) This was a cross-sectional study, which does not allow for the exploration of PD patients’ and their caregivers’ psychiatric disorders through the course of the disease and their caregiving. Therefore, further follow-up studies should be conducted to understand the precise nature of depression morbidity in PD patients and their caregivers, as well as any associated factors involved.

The clinical implications of this study are: (1) The morbidity of depression in PD patients is more prevalent than the morbidity of depression in their caregivers. (2) Fatigue, resilience, and suicidal ideation might contribute to the depression severity of PD patients and their caregivers. (3) Patients’ suicidal ideation negatively influenced their caregivers’ severity of depression. (4) It is crucial that clinicians are aware of, and manage these contributing factors in PD patients and their caregivers in order to prevent these two groups from worsening each other’s depression. Future studies are warranted to elucidate whether treatment for depression in both PD patients and their caregivers is beneficial for them both.

## Methods

### Participants

This study used a cross-sectional design with consecutive sampling. Participants were recruited from the neurology ward or neurology outpatient clinic at a general hospital from August 2018 to May 2020. Inclusion criteria of patients: (1) Individuals have been diagnosed with PD by an expert neurologist; (2) Individuals are able to understand the study procedure and can provide the written informed consent. Exclusion criteria of patients: (1) Individuals with a diagnosis of delirium, or atypical parkinsonism (e.g. Dementia with Lewy bodies, progressive supranuclear palsy, multiple system atrophy, corticobasal syndrome) or secondary parkinsonism; (2) Individuals who are too weak to complete the questionnaire or clinical interview.

Inclusion criteria of caregivers: (1) Individuals are patient’s principal caregivers. Principal caregivers are defined as “family members who living with the patients and taking care of their daily needs”; (2) Individuals are able to understand the study procedure and can provide written informed consent. Exclusion criteria of caregivers: Individuals who are too weak to complete the questionnaire or clinical interview.

In total, 192 PD patients and caregivers were invited to take part in this study initially; data collection was completed for 175 PD patients and caregivers (response rate: 91.1%). Among all the PD patients, three of them were too weak to complete the questionnaire or clinical interview, and 14 refused to partake in the interview. Among the 175 caregivers, 133 (76%) were spouses, 32 (18.3%) were children, and 10 (5.7%) were parents, siblings, or friends.

### Assessments

#### Unified Parkinson's Disease Rating Scale (UPDRS) (scores range from 0 to 144)

The UPDRS is the most commonly used scale in the clinical study of PD, and is used to follow the longitudinal course of PD^41^. The UPDRS is made up of the following sections: Part I: evaluation of behavioral problems such as intellectual decline, hallucinations, and depression; Part II: self-evaluation of activities of daily living (ADLs); Part III: clinician-scored monitored motor evaluation; Part IV: complications of therapy; Part V: Hoehn and Yahr staging of severity of PD^42^.

#### Fatigue Severity Scale (FSS) (scores range from 9 to 63)

The Fatigue Severity Scale (FSS) is a nine-item scale that measures the impact of fatigue on motivation, exercise, physical functioning, and interference with professional, familial, and social life^41^. The FSS is widely used to identify features of fatigue related to medical conditions, including multiple sclerosis, systemic lupus erythematosus, cancer, and PD^43^. The FSS can be applied to examine fatigue severity in caregivers^44^. The Chinese version of the FSS was validated for assessing fatigue-related impairment in Chinese-speaking persons with major depressive disorder^45^.

#### Connor–Davidson resilience scale (CD-RISC) (scores range from 0 to 40)

The original Connor-Davidson Resilience scale (CD-RISC) was a self-reported 25-item scale with scores ranging from 0 to 4 per item. Higher scores mean greater resilience, i.e., a greater ability to cope with stress. An abbreviated version of the CD-RISC comprising 10 items was developed on the basis of factor analysis, providing a rapid and brief method to quantify resilience^46^. Good reliability and validity were confirmed among different populations and diseases^47^.

#### Beck hopelessness scale (BHS)

The Beck Hopelessness Scale is a 20-item, self-reported tool designed to measure three major aspects (affective, motivational and cognitive) of hopelessness: feelings about the future, loss of motivation, and expectations^48^. Scores range from 0 to 20, with higher scores indicative of higher levels of hopelessness and higher suicide risk. The Chinese version of the BHS was translated and validated in Taiwan^49^.

#### Mini international neuropsychiatric interview (MINI)

The MINI is a short, structured clinical interview which assists researchers in executing diagnoses of psychiatric disorders, especially depressive disorders and anxiety disorders, based on DSM-IV or ICD-10 criteria^50^. The MINI was designed for epidemiological studies and has achieved satisfactory levels of validity and reliability^51^. Approximately 15–20 min are needed to conduct the interview.

#### Hamilton depression rating scale (HAM-D)

It is used to probe mood, feelings of guilt, suicidal ideation, insomnia, agitation, or retardation, anxiety, weight loss, and somatic symptoms^52^. The HAM-D has been widely applied to assess the severity of depression, though it has been criticized for over-emphasis on neuro-vegetative symptoms. The HAM-D is administered by clinicians or researchers. The reliability and validity of the Chinese version of the 17-item HAM-D has been verified, and it can be used in clinical and research settings^53^.

### Procedures

Ethical approval was obtained from the human research ethics committee of Chang Gung Memorial Hospital (201702186B0). Study procedures were as follows: (1) Once our research assistant received a referral from the neurological ward or neurological outpatient clinic from the in-charge doctor, the research assistant went to the to contact the person with PD and his/her caregiver. After explaining the study procedure and aims, only those patients and their caregivers who agreed to sign an informed consent form were enrolled in the study. (2) Both the PD patients and their caregivers received the BHS, FSS, CD-RISC, and the MINI. Additionally, PD patients received the UPDRS assessment. (3) The MINI was used by two staff psychiatrists (Dr. Y. Lee and Dr. YJ. Chiou) to reach a psychiatric diagnosis. (4) The HAMD-D was administered by Dr. Y. Lee and Dr. YJ. Chiou to evaluate depression severity. (5) Our trained research assistant collected the patients’ demographic and clinical data (including UPDRS), and the caregivers’ demographic data and clinical rating scales, including the FSS, BHS, CD-RISC. (6) Dr. Y. Lee and Dr. YJ. Chiou discussed the psychiatric diagnosis in the first three sessions of the research meeting to reach a psychiatric diagnostic consensus.

### Statistical analyses

Descriptive and inferential statistics were analyzed using SPSS for Windows V. 16.0. The non-parametric test (Mann–Whitney U test) was suitably performed because the number of depressive patients and caregivers were far less than the number of non-depressive patients and caregivers. Descriptive statistics (chi-square and Mann–Whitney U tests) were used first to test the difference in demographic data and then to test the clinical characteristics between subjects with and without depressive disorder. To determine the impact of fatigue, resilience, and suicidal ideation on patients’ and caregivers’ depression, we demonstrated actor and partner effects by using the actor–partner interdependence model (APIM) with a distinguishable dyads regression model^32^. The APIM is a model that integrates a conceptual view of interdependence with the appropriate statistical techniques for measuring and testing dyadic relationships by a distinct regression model^54^. The APIM was assessed using structural equation modeling (SEM)^54^. The SEM statistical program was analyzed using SPSS Amos 24.0.

### Ethical approval

All procedures performed in studies involving human participants were in accordance with the Declaration of Helsinki (1964) and its later amendments or comparable ethical standards. We obtained informed consent in writing from all individuals with PD and their caregivers.
